# Epidemiology of Intestinal Parasitic Infections Among Sengota Primary Schoolchildren in Dendi District, West Shoa Zone, Ethiopia

**DOI:** 10.1155/japr/4066213

**Published:** 2026-04-03

**Authors:** Teka Tolera, Damtew Bekele, Tilahun Yohanes, Solomon Tesfaye

**Affiliations:** ^1^ Department of Biology, College of Natural and Computational Sciences, University of Gondar, Gondar, Ethiopia, uog.edu.et; ^2^ Department of Biology, College of Natural and Computational Sciences, Ambo University, Ambo, Ethiopia, ambou.edu.et

**Keywords:** Dendi district, formol-ethyl acetate concentrate techniques, prevalence, risk factors, schoolchildren

## Abstract

**Background:**

Intestinal parasitic infections (IPIs) continue to pose serious public health issues in developing countries, including Ethiopia.

**Objective:**

This study is aimed at determining the prevalence of IPIs and associated risk factors among children at Sengota Primary School in the Dendi District, Oromia Region.

**Methods:**

A cross‐sectional study was conducted from October to November 2020. Sociodemographic data and potential risk factors for the occurrence of IPIs were collected using a structured, pretested questionnaire. Stool samples were gathered to detect parasites and examined using direct wet mount and formol‐ethyl acetate concentration methods. Data analyses were performed using SPSS statistical software, Version 20.

**Results:**

The overall prevalence of intestinal parasites among children in the study area was 25.5% (103/404) with 95% CI (21.0%–29.5%). The prevalence rates for protozoa, helminths, and polyparasitism were 14.1% (57/404), 8.2% (33/404), and 3.2% (13/404), respectively. The most prevalent protozoan parasites were *Giardia duodenalis,* affecting 11.1% (45/404), whereas the prevailing helminth was *Ascaris lumbricoides,* accounting for 5.0% (20/404). Washing hands sometimes before meals and after defecations (adjusted odds ratio, AOR: 11.148, 95% CI = 4.666, 26.071), never wash hands before meals and after defecations (AOR: 9.290, 95% CI = 0.611–141.321), using river water for drinking purposes (AOR = 7.298, 95% CI = 2.076–25.595), the use of groundwater as a source of drinking purposes (AOR = 28.866, 95% CI = 8.288–100.538), defecating in open field (AOR: 37.306, 95% CI = 4.534–306.930) were significantly associated with IPIs in the present study.

**Conclusions:**

The study findings indicate that IPIs are common among children at Sengota Primary School. Contributing factors include poor hygiene practices, limited access to safe drinking water, and insufficient latrine facilities. To effectively reduce the incidence of these infections, it is essential to enhance public health initiatives and establish regular deworming programs in schools.

## 1. Introduction

Intestinal parasitic infections (IPIs) are widespread, particularly in tropical and subtropical regions, making them common diseases in developing nations with poor socioeconomic conditions and low living standards [1]. Ethiopia carries one of the largest burdens of IPIs, accounting for 8% of all soil‐transmitted helminth (STH) infections globally [2]. Epidemiological data show that school‐aged children are more susceptible to IPIs and are likely to experience more severe infections compared with other age groups [3].

IPIs are highly prevalent among school‐aged children, with rates ranging from 66.7% to 83.8% [4]. This widespread occurrence creates significant public health challenges [5], reducing quality of life and potentially causing growth and cognitive delays, especially in children [6]. In areas with poor sanitation or where practices such as open defecation are common, parasite eggs can contaminate the soil, leading to infections through the consumption of contaminated food or contact with contaminated hands [7]. Several factors contribute to the high prevalence of these infections in tropical and subtropical regions. These factors include rising population density, inadequate sanitation, poor public health measures, insufficient restroom facilities, contaminated food and water, malnutrition, low host resistance, and environmental changes [8]. In lower‐income countries, *Entamoeba histolytica/dispar* and *Giardia duodenalis* are the leading causes of diarrheal diseases. These infections negatively affect health at both individual and societal levels due to poverty, a lack of health education, and limited access to clean water and healthcare services [9].

Parasitic infections are so common in Ethiopia that some parasites are often regarded as a “normal” part of the body [10]. This viewpoint indicates that many people live in areas without basic amenities and are unaware of the risks associated with exposure to IPIs. Key risk factors contributing to the transmission of IPIs among school‐aged children in Ethiopia include poor personal hygiene, a lack of safe drinking water, open defecation, and inadequate waste disposal practices [11–16].

Numerous epidemiological studies [17, 18] have been conducted to assess the prevalence of IPIs among schoolchildren in Ethiopia. However, to our knowledge, this study is the first to investigate the prevalence of IPIs and their associated risk factors in the Dendi District of the West Showa Zone, Oromia. The primary objective of the current study was to determine the prevalence and risk factors of IPIs among schoolchildren at Sengota Primary School in the Dendi District of the Oromia Region.

## 2. Materials and Methods

### 2.1. Study Area

**Figure 1:**
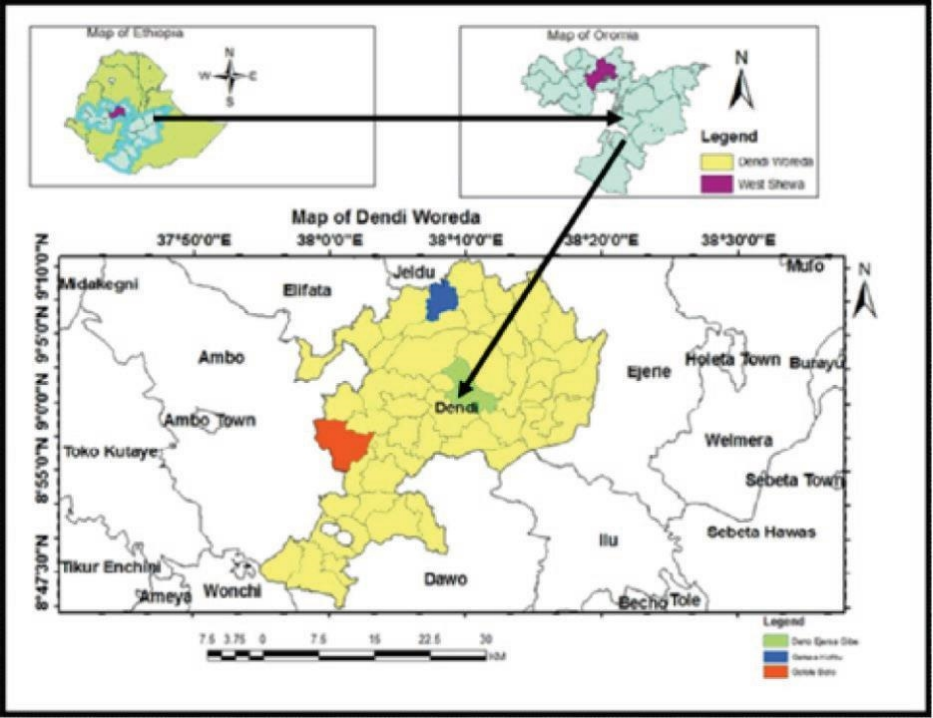
Map of the study area (Source: Ethio‐GIS and CSA Data).

The study area is located in the Dendi District of the West Showa Zone in the Oromia Region (Figure 1). Dendi District is situated 83 km west of Addis Ababa, the capital city of Ethiopia. It has a total population of 200,715, which includes 98,350 females and 102,365 males. The district covers an area of 1296.12 km^2^ and consists of 35 rural kebeles (the small admistrative unit in Ethiopia), four urban kebeles, and a total of 39 kebeles. Dendi District lies between latitudes 8°43 ^′^30“N and 9°17 ^′^0”N and longitudes 37°47 ^′^0“E and 38°20 ^′^0”E. The elevation ranges from 1440 to 3260 m above sea level (a.s.l).

The climate in Dendi District is tropical, with an average annual rainfall of 1140 mm and a mean maximum temperature of 23.3°C, whereas the mean minimum temperature is 9.6°C. The district features diverse topography, including distinct agroecological zones known locally as Dega (cool highlands) and Woina Dega (temperate mid‐altitudes).

Dendi District has 53 schools, including 47 primary schools for Grades 1 through 8, four secondary schools, a college of technology and vocational training, and a preparatory school located in Ginchi Town. The local healthcare network consists of one primary hospital, seven health centers, and 39 health posts, all of which provide preventive, promotional, and curative services. For many residents, mixed farming is the primary source of income. Agriculture typically employs traditional farming methods and relies heavily on seasonal rains. The main crops cultivated in the district include barley, teff, potatoes, wheat, and sorghum. Additionally, the district has a significant number of domesticated animals, including horses, mules, sheep, cattle, and goats.

### 2.2. Study Design and Period

This cross‐sectional study was conducted among primary schoolchildren in Sengota, located in the Dendi District of West Shoa, Ethiopia, from October to November 2020.

### 2.3. Inclusion and Exclusion Criteria

#### 2.3.1. Inclusion Criteria

The study involved students from Sengota Primary School whose parents or guardians granted informed consent for their participation.

#### 2.3.2. Exclusion Criteria

The study excluded students who had received anthelmintic or antiprotozoan treatment within 2 months prior to the study, those currently on such medications, and students outside the targeted age and grade ranges.

### 2.4. Sample Size Determination

A total of 404 participants were selected from students in Grades 1–8 at Sengota Primary School. The sample size was calculated using the Daniel [19] prevalence formula, with participants chosen proportionally based on the total school population. The calculation is detailed below:

N=Z2P1−PD2

where *N* = the total sample size number of study participants in the study. *Z* ^2^ = standard value. *P* = expected prevalence of IPIs in the study area. *D* = marginal error 5*%*, at 95% confidence interval (CI).

As there were no prior reports of IPIs in the study area, the sample size was calculated based on an assumed 50% prevalence to ensure maximum sample size. Using the formula for a single proportion with a 95% CI (*Z* = 1.96) and a 5% margin of error (*d* = 0.05), the initial sample size was determined to be 384. To account for a 5% nonresponse rate, the final sample size was increased to 404 students. Sengota Primary School, which had a total enrollment of 1886 children, was purposively selected from the primary schools in the Dendi District for this study.

### 2.5. Sampling Procedure

**Figure 2:**
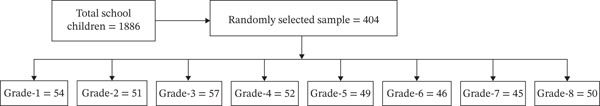
The stratification of the study population using source [5].

A stratified systematic sampling technique was used to select the 404 study participants. First, the student population was divided into strata based on grade level (Grades 1 through 8). Next, a proportional allocation method determined the number of students needed from each grade, ensuring that the sample accurately reflected the size of each grade level. Finally, participants were selected from the class rosters of each grade using systematic random sampling. The distribution of the sampled students across grades is illustrated in Figure 2.

### 2.6. Data Collection and Processing

#### 2.6.1. Sociodemographic and Risk Factor Data

Data on sociodemographic characteristics and risk factors for IPIs were collected through a structured questionnaire administered to both the children and their parents. Parents provided information on household variables such as marital status, education level, and occupation. Children, with assistance from their guardians when needed, supplied information regarding their personal hygiene and behavioral risks. This included details on whether they wore shoes, kept their nails trimmed, and adhered to water, sanitation, and hygiene (WASH) practices.

The questionnaire was first developed in English and then translated into Afan Oromo. To ensure consistency in language, it was subsequently back translated into English. Data collection was carried out by two trained health professionals, each holding a Bachelor′s degree. They conducted interviews to ensure that all questions were clearly understood by the participants and their guardians.

#### 2.6.2. Parasitological Survey

A sufficient stool specimen was collected from each participant for parasitological analysis, utilizing both direct wet mount and formol‐ether concentration techniques. To detect motile trophozoites, a direct wet mount was performed at the Dendi District Health Center within 30 min of specimen collection. This immediate examination enabled prompt treatment for students who tested positive on‐site.

The remaining portions of the samples were preserved in 10% formalin and transported to the biology laboratory at the University of Gondar for further analysis. The formol‐ether concentration technique was used to enhance the sensitivity of the analysis, especially for detecting parasites present in low densities. In the final prevalence analysis, a participant was considered positive if parasites were detected using either the direct wet mount or the concentration technique.

##### 2.6.2.1. Direct Wet Mount Preparation.

Each participant provided approximately 4 g of fresh stool, which was collected in a clean, prelabeled plastic container. Following standard laboratory protocols for tropical countries [20, 21], direct wet mounts were prepared by emulsifying about 2 g of the specimen in 0.85% normal saline. To improve the visualization of internal structures, a second mount was created using Lugol′s iodine on the opposite side of the same slide. The prepared smears were systematically examined under a light microscope, first using 10× magnification for initial scanning and then 40× magnification for a detailed identification of protozoan cysts, trophozoites, and helminth ova or larvae [22].

##### 2.6.2.2. Formol‐Ether Concentration Technique.

The formol‐ether concentration technique was used on preserved stool specimens to improve the detection of parasites. Approximately 1 g of stool was mixed with 10 mL of 10% formalin to create a uniform suspension. Next, 7 mL of this suspension was filtered through double‐layer cotton gauze into a centrifuge tube. After adding 3 mL of diethyl ether, the tube was stoppered and shaken vigorously for 30 s while inverted. The mixture was then centrifuged at 3000 rpm for 5 min, resulting in the formation of four distinct layers: an ether layer, a plug of fecal debris, a formalin layer, and sediment. After carefully discarding the top three layers and removing any debris from the walls of the tube with a cotton swab, the sediment was resuspended in a drop of formalin or saline. Finally, a wet mount stained with Lugol′s iodine was prepared from the sediment and examined under a microscope at 10× and 40× magnification to identify helminth ova, larvae, and protozoan cysts [20].

### 2.7. Data Analysis

The data collected were entered, cleaned, and coded using SPSS Version 20.0 (SPSS Inc., Chicago, Illinois, United States). Descriptive statistics were used to summarize the sociodemographic characteristics and to describe the distribution of study variables. To examine the associations between infectious disease incidence (IPIs) and various risk factors, chi‐square (*χ* ^2^) tests and bivariate logistic regression analyses were conducted.

To control for potential confounders and to identify independent predictors of IPIs, variables with a *p* value of less than 0.25 from the bivariate analysis were included in a multivariable logistic regression model. In the final model, statistical significance was determined using a threshold of *p* < 0.05. The results were presented using odds ratios (OR) and 95% CIs to indicate the strength and precision of the associations.

### 2.8. Quality Control

To ensure the validity and reliability of the study, research assistants underwent extensive training before data collection began. The structured questionnaires were pretested to confirm their clarity and logical flow. Additionally, all instruments were translated into Afan Oromo and then back translated into English to maintain linguistic integrity. At the end of each data collection day, the questionnaires were reviewed for completeness and internal consistency.

Laboratory quality was upheld through rigorous microscopic protocols. To ensure the accuracy of the direct wet mount, slides were meticulously inspected for impurities. As a quality control measure, 10% of the stool specimens were randomly selected and re‐examined by a senior technologist. All slides were assessed independently by two laboratory professionals. In the event of discrepancies in the results, the slides were re‐evaluated through collaborative discussion and, if necessary, with input from a third senior expert until a consensus was reached.

### 2.9. Ethical Considerations

Ethical clearance was obtained from the Ethical Review Committee of the University of Gondar, College of Natural and Computational Sciences (Ref. No. CNCS/10.638‐25.5.2020). Additionally, permission to conduct the study was granted by the Dendi District Education and Health Offices. Prior to data collection, parents and participants were fully informed about the study′s objectives, procedures, and their right to withdraw at any time. Written informed consent was obtained from parents or guardians, whereas verbal assent was acquired from the children. To ensure the well‐being of participants, all students diagnosed with IPIs received free medical treatment from professional nurses at the Dendi District Health Center, in accordance with national treatment guidelines. All personal information and laboratory results were kept strictly confidential using anonymous coding.

## 3. Results

### 3.1. Sociodemographic Characteristic of Study Participant

The study enrolled 404 participants, consisting of 227 (56.2%) males and 177 (43.8%) females. In terms of age distribution, the 4–8 year age group was the most represented (48.5%), followed by those aged 9–13 (34.7%) and 14–18 (16.8%). Regarding educational and residential status, over half of the participants (53%) were in Grades 1–4, and the majority (67.2%) resided in urban areas. Additionally, 52.5% of the participants came from households with more than five members (Table 1).

**Table 1:** Sociodemographic characteristic of study participants attending at Sengota primary school, Dendi district, Oromia region, from October to November 2020.

Variables	Categories	Frequency	Percentage
Sex	Male	227	56.2
	Female	177	43.8

Age	4–8	196	48.5
	9–13	140	34.7
	14–18	68	16.8

Grade level	1–4	214	53.0
	5–8	190	47.0

Residence	Urban	271	67.2
	Rural	133	32.9

Family Size	1–5	194	48.0
	6 and above	210	52.0

### 3.2. Prevalence of IPI in Sengota Primary School

Out of the 404 school‐aged children examined for the presence of IPIs, the overall prevalence was found to be 25.5% (103/404; 95% CI: 21.0%–29.5%). Protozoan infections were more frequent (14.1%) than helminthic infections (8.2%). The study identified two protozoan and two helminth species in the collected stool samples. Regarding the intensity of infection, 22.3% (*n* = 90) of children had single infections, 2.9% (*n* = 12) had double infections, and 0.2% (*n* = 1) had a triple infection. Notably, among the double infections, seven cases involved coinfection with *G. duodenalis* and *Ascaris lumbricoides (A. lumbricoides*). Individually, *G. duodenalis* was the most prevalent parasite at 11.1% (45/404), followed by *A. lumbricoides* at 5.0% (20/404) and *E. histolytica/dispar* at 3.0% (12/404) (Table 2).

**Table 2:** Prevalence of IPIs in Sengota primary schoolchildren (*n* = 404), Dendi District, West Showa Zone, Oromia region, from October to November 2020.

Parasite Species identified	Frequency	Percentage
Protozoa		
*Giardia duodenalis*	45	11.1
*Entamoeba histolytica/dispar*	12	3.0
Total	57	**14.1**

Helminths		
*Ascaris lumbricoides*	20	5.0
*Hymenolepis nana*	13	3.2
Total	33	8.2

Mixed infection		
*G*.*d* *u* *o* *d* *e* *n* *a* *l* *i* *s* + *A*.*l* *u* *m* *b* *r* *i* *c* *o* *i* *d* *e* *s*	7	1.7
*G*.*d* *u* *o* *d* *e* *n* *a* *l* *i* *s* + *E*.*h* *i* *s* *t* *o* *l* *y* *t* *i* *c* *a*/*d* *i* *s* *p* *a* *r*	5	1.2
*G*.*d* *u* *o* *d* *e* *n* *a* *l* *i* *s* + *E*.*h* *i* *s* *t* *o* *l* *y* *t* *i* *c* *a*/*d* *i* *s* *p* *a* *r* + *H*.*n* *a* *n* *a*	1	0.3
Total	13	3.2

Overall parasite prevalence		
Single infection	90	22.3
Double infection	12	2.9
Triple infection	1	0.2
Total	103	25.5

*Note:* Bold value indicate higher prevalence.

### 3.3. Sociodemographic Characteristics and Prevalence of IPIs

The univariate logistic regression analysis of sociodemographic factors associated with IPIs is summarized in Table 3. In this study, none of the sociodemographic variables were found to be statistically significant. When considering place of residence, urban residents demonstrated a higher likelihood of infection compared with those living in rural areas (COR: 1.435; 95% CI: 0.875–2.353), although this trend did not reach statistical significance (*p* = 0.152). Additionally, children whose fathers worked as farmers were more than 2.5 times more likely to be infected compared with those whose fathers were merchants (COR: 2.536; 95% CI: 0.867–7.418). However, this association also did not meet the criteria for statistical significance (*p* = 0.089) (Table 3).

**Table 3:** Univariate analysis of sociodemographic characteristic and prevalence of intestinal parasitic infections at Sengota primary schoolchildren (*n* = 404) Dendi District, Oromia region, from October to November 2020.

Variables	Categories	Total examined *n* (%)	Negative *n* (%)	Positive *n* (%)	COR (95% CI)	*p* value
Sex	Male	227	167 (73.6)	60 (26.4)	1.110 (0.712, 1.761)	0.625
	Female	177	134 (75.7)	43 (24.3)		

Age	4–8	196	144 (73.5)	52 (26.5)	1.174 (0.616, 2.234)	0.626
	9–13	140	105 (75)	35 (25)	1.083 (0.050, 2.135)	0.817
	14–18	68	52 (76.5	16 (23.5)	1	

The parent residence	Urban	271	196 (72.3)	75 (27.7)	1.435 (0.875, 2.353)	0.152
	Rural	133	105 (78.9)	28 (21.1)	1	

Family size	1–5	194	143 (73.7)	52 (26.3)	1.084 (0.693, 1.695)	0.725
	6 and above	210	158 (75.2)	52 (24.8)	1	

Mother occupation	Farmer	153	115 (75.2)	38 (24.8)	0.943 (0.590, 1.516)	0.805
	Government employee	8	6 (75)	2 (25)	0.951 (0.187, 4.837)	0.952
	Merchant	8	6 (75)	2 (25)	0.951 (0.187, 4.837)	0.952
	House wife	235	174 (74)	62 (26)	1	

Father occupation	Farmer	361	265	96 (26.6)	2.536 (0.867, 7.418)	0.089
	Government employee	11	(73.4)	3 (27.3)	2.625 (0.484,14.235)	0.263
	Merchant	32	8 (72.7)	4 (12.5)	1	
			28 (77.5)			

Mother education	Primary school (1–8)	203	151 (74.4)	52 (25.6)	1.257(0.689, 2.2601)	0.445
		87	62 (71.3)	25 (28.7)	1.472 (0.747, 2.901)	0.264
	Secondary school (9–12) diploma and above	21	15 (71.40	6 (28.6)	1.460 (0.502, 4.249)	0.487
	No formal education	93	73 (78.5)	20 (21.5)	1	

Father education	Primary school (1–8)	157	116 (73.9)	41 (26.1)	1.074 (0.608, 1.896)	0.802
		110	82 (74.5)	28 (25.5)	1.038 (0.560, 1.922)	0.907
	Secondary school (9–12) diploma and above	32	24 (75)	8 (25)	1.013 (0.4–6, 2.528)	0.978
	No formal education	105	79 (75.2)	26 (24.8)	1	

### 3.4. Personal Hygiene and Sanitation Characteristics and Prevalence of IPIs

The relationship between personal hygiene, sanitation practices, and the prevalence of IPIs is summarized in Table 4. A multivariate logistic regression analysis identified several independent predictors of infection. These included the frequency of hand washing, the source of drinking water, and defecation habits.

**Table 4:** Univariate and multivariate analysis between personal‐hygiene and sanitation effects on prevalence of intestinal parasitic infections at Sengota primary schoolchildren (*n* = 404) Dendi wereda, Oromia region, from October to November 2020.

Variables	IPIs status		COR (95% CI)	*p* value	AOR (95% CI)	*p* value
	Negative (%)	Positive (%)				
Hand washing before meals and after defecations						
Regularly	129 (64.0)	7 (5.2)	Ref			
Sometimes	169 (20.0)	95 (36.0)	10.279 (4.613, 22.904)	*p* ≤ 0.001	11.148 (4.660, 26.671)	*p* ≤ 0.001
Never	4 (20.0)	12 (20.0)	4.571 (0.449, 46.449)	0.199	9.290 (0.611, 141.321)	0.109

Water source						
Tap water	90 (96.8)	3 (3.2)	Ref			
River water	143 (82.2)	31 (17.8)	6.503 (1.931, 21.898)	0.003	7.289 (2.076, 25.595)	**0.002**
Ground water	68 (49.6)	69 (50.4)	30.441 (9.187, 100.872)	*p* ≤ 0.001	28.866 (8.288, 100.538)	*p* ≤ 0.001

Habit of defecation						
Open field	164 (64.1)	92 (35.9)	6.987 (3.592, 13.588)	*p* ≤ 0.001	37.306 (4.534, 306.930)	*p* ≤ 0.001
Pit latrine	137 (92.7)	11 (7.4)	Ref		Ref	

swimming habit						
Sometimes	19 (79.2)	5 (20.8)	0.757 (0.275, 2.0829)			
Never	282 (74.2)	98 (25.8)	Ref	0.757	NA	

Waste disposal methods						
Burn	130 (88.4)	17 (11.6)	1.308 (0.157, 10.860)	0.804	22.203 (0.850, 530.147)	0.063
Open field	161 (65.4)	85 (34.6)	5.280 (0.865, 41.938)	0.116	3.894(0.287,52.806)	0.307
Bury	10 (90.9)	1 (9.1)	Ref			

Shoe wearing habit						
Always	118 (71.8)	42 (28.5)	1.294 (0.403, 4.173)	0.666		
Sometime	170 (76.6)	52 (23.4)	0.994 (0.311, 31.80)	0.992		
No	13 (76.5)	4 (23.5)	Ref			

Close contact with animals						
Always	223 (73.1)	82 (26.9)	1.532 (0.607, 3.869)	0.367	NA	
Sometimes	53 (77.9)	15 (22.1)	1.179 (0.409, 3.402)	0.760		
Never	25 (80.6)	6 (19.4)				

Finger trimming habit						
Always	17 (68.0)	8 (32.0)	3.294 (0.345, 31.490)	0.301	NA	
Sometimes	277 (74.7)	94 (25.3)	2.375 (0.288, 19.560)	0.421		
Never	7 (87.5)	1 (12.5)				

Washing fruit or vegetable before eating						
Always	95 (68.8)	43 (31.2)	1.554 (0.980, 2.461)	0.061	1.712 (0.941, 3.116)	0.078
Sometimes	206 (77.4)	60 (22.6)	Ref			

Personal hygiene						
Good	111 (77.1)	33 (22.9)	0.807 (0.502, 1.298)	0.807	NA	
Poor	190 (73.1)	70 (26.9)	Ref			

Food storage place						
Good	212 (76.5)	65 (23.5)	1.040 (0.644, 1.678)	0.874	NA	
Poor	89 (70.1)	38 (29.9)	Ref			

Abbreviations: 95% CI, 95% confidence interval; AOR, adjusted odds ratio; COR, crude odds ratio; NA, not applicable.

Participants who reported washing their hands only “sometimes” were found to be 11 times more likely to be infected compared with those who practiced regular hand washing (adjusted odds ratio (AOR): 11.148; 95% CI: 4.660–26.671; *p* < 0.001). Regarding water sources, individuals who consumed groundwater faced a significantly higher risk of infection (AOR: 28.866; 95% CI: 8.288–100.538; *p* < 0.001), as did those who drank river water (AOR: 7.289; 95% CI: 2.076–25.595; *p* = 0.002), compared with participants using tap water.

Moreover, the most significant predictor of infection was related to defecation practices. Individuals who practiced open‐field defecation were over 37 times more likely to contract IPIs than those who used pit latrines (AOR: 37.306; 95% CI: 4.534–306.930; *p* < 0.001) (see Table 4).

### 3.5. Potential Risk Factors Associated With *G. duodenalis*, *E. histolytica/dispar*, *A. lumbricoides*, and *Hymenolepis nana*

#### 3.5.1. Risk Factors for Protozoan Infections (*G. duodenalis* and *E. histolytica/dispar*)

The chi‐square analysis identified several significant environmental and behavioral drivers for *G. duodenalis* infection. Prevalence was notably lower among participants who practiced regular hand washing before meals. In contrast, those consuming well water had a significantly higher infection rate compared with tap water users (19.7% vs. 1.1%). Furthermore, open‐field defecation (*χ* ^2^ = 16.793, *p* < 0.001) and open waste disposal (*χ* ^2^ = 12.072, *p* < 0.001) were strongly associated with increased *G. duodenalis* prevalence. Furthermore, close contact with animals was also found to increase the prevalence of *G. duodenalis* in the study (Table 5).

**Table 5:** The association between potential risk factors and single species of IPIs identified at Sengota primary schoolchildren (*n* = 404), Dendi District, Oromia region, from October to November 2020.

Variables	Categories	Negative *n*(%)	Positive *n*(%)	*χ* ^2^	*p* value
*G. duodenalis*					
Hand washing before meals	Regularly	133 (98.5)	2 (1.5)	20.419	*p* < 0.001
	Sometimes	221 (83.7)	43 (16.3)		
	Never	5 (100)	0 (0)		

Water Source	Tap water	92 (98.9)	1 (1.1)	20.009	*p* < 0.001
	River	157 (90.2)	17 (9.8)		
	Ground water	110 (80.3)	27 (19.7)		

Habit of defecation	Open field	215 (84.0)	41 (16.0)	16.793	*p* < 0.001
	Pit latrine	144 (97.3)	4 (2.7)		

Waste disposal methods	Burn	140 (95.2)	7 (4.8)	12.072	0.02
	Open field	208 (84.6)	38 (15.4)		
	Burry	11 (100)	0 (0)		

Close contact with animals	Always	266 (87.2)	39 (12.8)	5.091	0.075
	Sometimes	62 (91.2)	6 (8.8)		
	Never	31 (100)	0 (0)		

*E. histolytica/dispar*					
Water Source	Tap water	93 (100.0)	0 (0)	18.477	*p* < 0.001
	River	173 (98.4)	1 (0.6)		
	Ground water	126 (92.6)	11 (8.0)		

Habit of defecation	Open field	245 (95.7)	11 (4.3)	4.267	0.039
	Pit latrine	147 (99.3)	1 (0.7)		

Close contact with animals	Always	299 (98)	6 (2.0)	6.394	0.041
	Sometimes	65 (95.6)	3 (4.4)		
	Never	29 (90.3)	3 (9.7)		

*A. lumbricoides*					
Water Source	Tap water	92 (98.9)	1 (1.1)	10.877	0.040
	River	170 (97.7)	4 (2.3)		
	Ground water	125 (91.2)	12 (8.8)		

Personal‐hygiene	Good	144 (100)	0 (0)	9.829	0.02
	Poor	343(93.5)	17 (6.5)		

Washing fruit or vegetable before eating	Sometimes	199 (94.3)	10 (7.2)	4.801	0.028
	Always	259 (97.4)	7 (2.6)		

*H. nana*					
Sex	Male	199 (94.3)	11 (5.2)	5.167	0.049
	Female	161 (98.8)	2 (1.2)		

Hand washing before meals	Regularly	125 (95.2)	1 (0.8)	8.134	0.044
	Sometimes	231 (95.1)	11 (4.5)		
	Never	4 (80.0)	1 (20.0)		

Water Source	Tap water	93 (100)	0 (0)	13.446	0.002
	River	171 (98.3)	3 (1.7)		
	Ground water	126 (92)	11 (8.0)		

Habit of defecation	Open field	243 (94.9)	13 (5.1)	5.422	0.020
	Pit latrine	147 (99.3)	1 (0.7)		

For *E. histolytica/dispar*, higher prevalence was significantly associated with well water consumption (*χ* ^2^ = 18.477, *p* < 0.001) and open‐field defecation (*χ* ^2^ = 20.009, *p* < 0.001). Interestingly, and in contrast to *G. duodenalis*, *E. histolytica/dispar* rates were highest among participants with no animal contact, suggesting a primarily anthroponotic (human‐to‐human) transmission route in this study area.

#### 3.5.2. Risk Factors for Helminthic Infections (*A. lumbricoides* and *H. nana*)

The prevalence of *A. lumbricoides* was highest among those using ground water (8.8%). However, unlike the protozoan infections, the risk of *A. lumbricoides* was significantly mitigated by maintaining good personal hygiene and consistently washing fruits and vegetables before consumption (Table 5).

Finally, *H. nana* infection showed a statistically significant relationship with hand washing habits, water source, and defecation habits (*p* < 0.05). The highest prevalence was observed among participants who neglected hand washing before meals, relied on ground water, and practiced open‐field defecation (Table 5).

## 4. Discussion

The present study found an overall prevalence of IPIs at 25.4%. This rate is similar to findings from Birbir Town (27.1%) [5], Bahir Dar (24.4%) [22], and East Arsi (27.1%) [23]. However, it is significantly lower than reports from the Democratic Republic of São Tomé and Príncipe (64.7%) [3], Nigeria (86.2%) [24], Sudan (56.9%) [25], and the Sasiga district of Southwest Ethiopia (62.4%) [14]. This relatively lower prevalence may be attributed to improved personal hygiene practices among the study participants. These practices include regularly wearing shoes, washing fruits and vegetables, and maintaining good nail hygiene, all of which are known to be protective against intestinal parasites [15, 24].

In contrast, this prevalence is higher than what was reported in Debre Markos Town, Northwest Ethiopia (12%) [26], Saudi Arabia (12%) [27], and Kenya (16.4%) [28]. This discrepancy is likely attributable to differences in climatic conditions, environmental sanitation, and the socioeconomic status of parents in each country [25, 29–31].

In the study area, infections caused by protozoa were more common than those caused by helminths, with rates of 14.1% and 8.2%, respectively. This trend is consistent with findings from studies conducted in northwest Ethiopia [32], Burkina Faso (84.7%) [33], and Iran (32.3%) [34]. The higher prevalence of protozoan infections may be attributed to various environmental and behavioral factors, including poor hand hygiene before meals, consumption of untreated groundwater, open‐field defecation, proximity to livestock, and inadequate waste management. These conditions promote fecal–oral transmission [35]. Furthermore, mass drug administration programs that primarily target STHs may have selectively reduced helminth infections. As a result, protozoan parasites such as *G. duodenalis* and *E. histolytica* remain untreated, as these organisms are not affected by the commonly used anthelmintic medications [36].

Although protozoa were the most commonly identified organisms in this study, previous research conducted in southern and northwestern Ethiopia has reported a higher prevalence of helminthic infections [5, 32]. This indicates that local behaviors and environmental conditions significantly influence infection rates. In the study area, better adherence to preventive measures—such as consistently wearing shoes, keeping fingernails trimmed, and storing food safely—may have reduced exposure to STHs and interrupted fecal–oral transmission pathways [15, 32].

Single infections were the most common, accounting for 22.27% of cases. Double infections occurred in 2.97% of cases, whereas triple infections were quite rare, at 0.25%. These rates are lower than those reported in Jawi town [15] and Bahir Dar [37]. The differences may be attributed to variations in laboratory diagnostic sensitivity, levels of environmental contamination, and geographical and climatic conditions that influence parasite survival.

Sociodemographic factors, such as age, sex, and parental occupation, did not reveal any statistically significant associations with the overall prevalence of IPIs. However, there was an elevated crude odds ratio (COR: 2.5) among children of farmers, indicating a slightly increased risk for this group. This heightened risk may be linked to greater exposure to contaminated soil, animal feces, and agricultural environments, all of which are recognized as risk factors for IPIs [2]. The lack of statistical significance may suggest that environmental contamination is widespread and affects children across different demographic groups.

Among the individual parasites identified, *G. duodenalis* was the most prevalent species, accounting for 11.1% (*n* = 45) of the cases. This finding is consistent with previous research conducted in Jawi, Northwest Ethiopia [15]. The high prevalence of this parasite can be attributed to its low infectious dose, which ranges from 10 to 100 cysts, and its ability to survive in the environment for weeks in both soil and water. Additionally, *G. duodenalis* shows significant resistance to common chemical disinfectants, such as chlorine. This resistance facilitates transmission through unprotected water sources, which were identified in this study [38].

The second most common parasite identified was *A. lumbricoides*, which had a prevalence of 5% (*n* = 20). This rate is comparable with that reported in Colombia, which is 5.1% [6]. The persistence of *A. lumbricoides* can be attributed to the unique lipoprotein shell of its eggs, which makes them extremely resistant to desiccation and chemical exposure. This biological durability allows the eggs to remain viable in the soil for extended periods, leading to potential infections through contaminated hands or unwashed produce [39].

Clear species‐specific risk patterns were observed in the study. Irregular hand washing before meals was strongly associated with *G. duodenalis* infection (*p* < 0.001), which aligns with its established fecal‐oral transmission route [40, 41]. Additionally, the prevalence of *G. duodenalis*, *E. histolytica/dispar*, *A. lumbricoides,* and *H. nana* significantly increased when using unsafe drinking water sources, highlighting the critical role of water quality in the transmission of these parasites [38]. The analysis also revealed that sex was significantly linked only to *H. nana* infection, with higher prevalence observed among males. This may reflect gender‐related behavioral exposure differences, such as increased outdoor activities and lower compliance with hygiene practices [42]. Furthermore, close contact with animals was associated with *E. histolytica/dispar*, suggesting environmental contamination through shared water sources or household surroundings rather than direct zoonotic transmission [43].

Open‐field defecation, unsafe drinking water sources, and poor hand washing practices before meals and after using the toilet were identified as significant independent predictors of IPIs (*p* < 0.05). Children who did not regularly wash their hands before meals were over 11 times more likely to become infected (AOR = 11.148). In contrast, consistent hand washing after defecation offered protective benefits. Furthermore, children from households practicing open‐field defecation were 37 times more likely to acquire an infection (AOR = 37.306), indicating limited access to latrines and a lack of awareness regarding transmission routes. The use of river water or unprotected groundwater also increased the risk of infection due to contamination from human and animal waste. These findings are consistent with those from Delgi [44] and align with broader evidence related to WASH [45].

This study has several limitations. The cross‐sectional design makes it impossible to establish causal relationships. Furthermore, the prevalence of infections may be underestimated because techniques such as Mini‐FLOTAC, Baermann, and molecular DNA detection were not used. The absence of antigen tests also means that we could not differentiate between *E. histolytica* and *E. dispar*. Lastly, self‐reporting on sanitation and hygiene variables may have introduced social desirability bias or recall bias.

## 5. Conclusions

The findings of this study reveal that IPIs remain a significant public health challenge among school‐aged children, with an overall prevalence of 25.5%. The study highlights a shift in parasitic burden, where protozoan infections—specifically *G. duodenalis*—have become more prevalent than helminthic infections.

The most critical drivers of infection identified were open‐field defecation, the use of unprotected groundwater, and inconsistent hand washing. The exceptionally high odds of infection associated with open defecation (AOR: 37.306) and groundwater use (AOR: 28.866) indicate that environmental contamination is the primary pathway for transmission. Although sociodemographic factors did not show statistical significance, the overwhelming impact of sanitation and hygiene practices suggests that the risk of IPIs is universal across the study population due to shared environmental exposures.

## Author Contributions

T.T.: identified the research problem, collected and analyzed the data, and participated in the draft and final write‐up of the manuscript. D.B. and T.Y.: collected and analyzed the data, and participated in the draft and final write‐up of the manuscript. S.T.: identified the research problem, processing and data analysis, participated in the draft and final write‐up of the manuscript.

## Funding

No funding was received for this manuscript.

## Conflicts of Interest

The authors declare no conflicts of interest.

## Data Availability

The findings of this study were generated from the original data collected during the study period and analyzed based on the stated methods and materials. The original data used to support the findings of this study will be available at any time upon request.
